# Functional deficit in children with congenital heart disease undergoing surgical correction after intensive care unit discharge

**DOI:** 10.5935/0103-507X.20200042

**Published:** 2020

**Authors:** Eloisa da Rosa Schunck, Camila Wohlgemuth Schaan, Gabriela Alves Pereira, Nathália Vieira Rosa, Tatiana Coser Normann, Claudia Pires Ricachinevsky, Caroline Tozzi Reppold, Renata Salatti Ferrari, Janice Luisa Lukrafka

**Affiliations:** 1 Universidade Federal de Ciências da Saúde de Porto Alegre - Porto Alegre (RS), Brasil.; 2 Serviço de Fisioterapia, Hospital de Clínicas de Porto Alegre, Universidade Federal do Rio Grande do Sul - Porto Alegre (RS), Brasil.; 3 Unidade de Terapia Intensiva Pediátrica, Hospital da Criança Santo Antônio, Irmandade Santa Casa de Misericórdia de Porto Alegre - Porto Alegre (RS), Brasil.

**Keywords:** Intensive care units, pediatric, Heart defect, congenital, Thoracic surgery, Risk factors, Child, Unidades de terapia intensiva pediátrica, Cardiopatias congênitas, Cirurgia torácica, Fatores de risco, Criança

## Abstract

**Objective:**

To evaluate the functional status of pediatric patients undergoing congenital heart surgery after discharge from the intensive care unit, and to evaluate the correlations among clinical variables, functional status and surgical risk.

**Methods:**

Cross-sectional study including patients aged 1 month to less than 18 years undergoing congenital heart surgery between October 2017 and May 2018. Functional outcome was assessed by the Functional Status Scale, surgical risk classification was determined using the Risk Adjustment for Congenital Heart Surgery-1 (RACHS-1), and clinical variables were collected from electronic medical records.

**Results:**

The sample comprised 57 patients with a median age of 7 months (2 - 17); 54.4% were male, and 75.5% showed dysfunction, which was moderate in 45.6% of the cases. RACHS-1 category > 3 was observed in 47% of the sample, indicating higher surgical risk. There was a correlation between functional deficit and younger age, longer duration of invasive mechanical ventilation and longer intensive care unit stay. Moreover, greater functional deficit was observed among patients classified as RACHS-1 category > 3.

**Conclusion:**

The prevalence of functional deficit was high among children and adolescents with congenital heart disease after cardiac surgery. Higher surgical risk, longer duration of invasive mechanical ventilation, longer intensive care unit stay and younger age were correlated with worse functional status.

## Abstract (pt)

**Objetivo:**

Avaliar a funcionalidade de pacientes pediátricos submetidos à correção cirúrgica de cardiopatia congênita após a alta da unidade de terapia intensiva e as possíveis correlações com variáveis clínicas e risco cirúrgico.

**Métodos:**

Estudo transversal, que incluiu crianças entre 1 mês e 18 anos incompletos, que realizaram cirurgia para correção de cardiopatia congênita, no período de outubro de 2017 até maio de 2018. A avaliação da funcionalidade foi realizada por meio da *Functional Status Scale*, a avaliação do risco cirúrgico se deu pelo *Risk Adjustment* for Congenital Heart Surgery-1 (RACHS-1), e as variáveis clínicas foram obtidas do prontuário eletrônico.

**Resultados:**

A amostra foi composta de 57 crianças, com mediana de idade de 7 (2 - 17) meses, sendo 54,4% do sexo masculino. Dentre as crianças, 75,5% apresentaram alteração na funcionalidade, e 45,6% delas tiveram disfunção moderada. Cerca de 47% da amostra apresentou classificação RACHS-1 > 3, indicando maior risco cirúrgico. Maior déficit funcional foi associado a crianças mais novas, com maior duração da ventilação mecânica invasiva e do tempo de internação na unidade de terapia intensiva. Além disso, maior grau de disfunção foi observado entre aqueles classificados com RACHS-1 > 3.

**Conclusão:**

A prevalência de disfunção foi elevada em crianças e adolescentes com cardiopatia após cirurgia cardíaca. Maior risco cirúrgico, duração da ventilação mecânica invasiva, permanência na unidade de terapia intensiva e os mais jovens apresentaram associação com pior desempenho funcional.

## INTRODUCTION

Congenital heart defects are structural heart defects; they are the most common congenital abnormality.^(1)^ They have a prevalence of approximately 9.1 per 1,000 live births^(1)^ and have a negative impact on healthcare systems, as they are associated with high rates of associated chronic diseases and motor development delays and an increased number of annual medical visits.^(2)^ In most cases, treatment includes surgical correction with consequent admission to the pediatric intensive care unit (ICU).^(3)^ With the technological advances in the ICU setting, the mortality rate has decreased considerably in recent decades, but there has been an exponential increase in perceived morbidity after hospital discharge.^(3,4)^

Cardiac surgery has intrinsic risks due to the large number of anatomical abnormalities in these patients, which require highly complex and specific surgical techniques.^(5,6)^ The Risk Adjustment for Congenital Heart Surgery (RACHS-1) method has been reliably used to assess the surgical risk and likelihood of death in these patients, with evidence showing a direct association with postoperative functional outcomes.^(5,6)^ However, this risk assessment method does not consider factors inherent to pediatric heart surgery or to the postoperative period, such as gestational age, nutritional status, use of extracorporeal circulation (ECC) and invasive mechanical ventilation (IMV)^(7-9)^ and length of hospital stay.^(9-11)^

Interventions performed during hospitalization^(12-14)^ can cause deficits in the overall functional status of children.^(3,14-16)^ Studies have shown that functional decline is directly influenced by clinical variables^(3,17)^ and by risks inherent to the medical intervention and the underlying disease.^(15)^

Considering the presence of functional decline, the Functional Status Scale (FSS) was developed for use with children aged between 1 month and less than 18 years to measure functional status in activities of daily living (ADL) based on the adaptive behavior concept.^(18)^ The FSS was recently validated for the Portuguese language,^(19)^ and recent studies using this scale have shown functional decline after discharge from the pediatric ICU, with a prevalence ranging from 4.6%^(17)^ to 82%.^(14)^ Factors such as age, primary dysfunctional system, length of hospital stay and duration of IMV have a direct influence on the onset of deficits.^(14,15,17)^

Studies of functional outcomes after pediatric ICU stay have gained prominence recently, but they have include patients with a wide range of illnesses.^(20-22)^ In Brazil, studies on functional deficits in specific illnesses are scarce,^(14)^ making it difficult to identify potential risk groups and factors that may be associated with worsened functional status after discharge from the pediatric ICU.

The primary objective of the present study was to evaluate the functional status of pediatric patients after discharge from ICU who undergoing cardiac surgery and to determine the possible correlations with clinical variables and surgical risk. The secondary objective was to compare clinical variables and surgical risk among individuals with different degrees of impairment on the FSS-Brazil.

## METHODS

A cross-sectional analytical study was conducted at *Hospital da Criança Santo Antônio, Irmandade da Santa Casa de Misericórdia* hospital complex (ISCMPA), from June to August 2018. The study was approved by the Research Ethics Committee of *Hospital da Criança Santo Antônio* - *Santa Casa*/Rio Grande do Sul, under opinion 2.025.679.

The population comprised all children undergoing surgery to correct congenital heart disease between October 2017 and May 2018. Children of both sexes aged between 1 month and less than 18 years who underwent a surgical procedure for congenital heart disease correction included in the RACHS-1 and who were admitted to the pediatric ICU after the procedure for longer than 24 hours were included. Parents or guardians who agreed to their children’s participation in the study signed an Informed Consent Form. Patients who were readmitted to the pediatric ICU within less than 24 hours were excluded.

The clinical variables assessed included age at discharge from the pediatric ICU (months), weight (kg), height (cm) and Z-score (at discharge from the pediatric ICU), gestational age (weeks), sex, birth weight (kg), surgery duration, duration of ECC (minutes), length of stay in the pediatric ICU (during the pre- and postoperative periods), duration of IMV (in the pre- and postoperative periods), duration of noninvasive mechanical ventilation (in the pre- and postoperative periods), postoperative complications, presence of comorbidities and clinical diagnosis.

The World Health Organization (WHO) software WHO Anthro was used to calculate the Z-scores of patients up to 60 months old, and WHO Anthro Plus was used for patients over 61 months of age; the “anthropometric calculator” module of both programs was used. The analyzed Z-score corresponded to the body mass index (BMI) for age of the entire study sample.^(23,24)^

Postoperative complications were defined as unfavorable clinical events that occurred after the surgical procedure that were not present during the preoperative period, such as sepsis, atelectasis, pneumonia, renal and respiratory system abnormalities, and other complications. To identify prior comorbidities, the presence of diseases before the surgical procedure was analyzed.

Surgical risk was assessed using the RACHS-1 method, which assesses the risk of mortality based on the surgery the patient underwent. It was developed for use in children (ages zero to less than 18 years) undergoing surgery for congenital heart disease. The RACHS-1 is composed of six categories (1 - 6), and the higher the category, the greater the risk to which the child is exposed.^(6)^ In cases in which more than one procedure was performed, the highest risk category was used. In the present study, we categorized the RACHS-1 into categories 1, 2, 3 and > 3.^(25)^

Functional status was assessed within 48 hours after discharge from the pediatric ICU using the FSS-Brazil, which consists of six domains of functioning: mental status, sensory, communication, motor function, feeding and respiration. Each domain is scored from 1 to 5 (normal function and mild, moderate, severe and very severe dysfunction), resulting in final scores ranging from 6 to 30. The higher the score is, the worse the functional status of the patient.^(18)^ For the present study, the outcome was analyzed in two distinct ways: we first grouped the scores into levels of dysfunction, i.e., no dysfunction (6 - 7), mild dysfunction (8 - 9), moderate dysfunction (10 - 15), severe dysfunction (16 - 21) and very severe dysfunction (> 21), a classification method used in previous studies.^(14,15)^ Secondly, we dichotomized functional status into adequate function/mild dysfunction and moderate/severe dysfunction.

Information on the surgical procedure and patient clinical variables were collected by the responsible investigator from the electronic records in the Tasy system used at *Santa Casa* Hospital Complex. There was no investigator blinding to the RACHS-1 category. Functional status was assessed using the FSS-Brazil by an investigator who was blinded to the objectives and variables of the present study.

### Statistical analysis

Data are presented as the median and interquartile range for continuous variables and as relative and absolute frequencies for categorical variables. Analysis of the correlations between continuous variables and the functional score was performed using the Spearman test. Values of 0.30 - 0.50 (-0.30 - -0.50) were indicative of weak correlations; 0.50 - 0.70 (-0.50 - -0.70) indicated moderate correlations; and 0.70 - 0.90 (-0.70 - -0.90) indicated strong correlations.^(26)^ To evaluate the differences between the functional deficit groups (adequate function/mild dysfunction *versus* moderate/severe dysfunction), the Mann-Whitney U test was used for continuous variables, and the chi-square test was used for categorical variables. The statistical software Stata 14.0 was used, and a significance level of 5% was adopted.

## RESULTS

The total sample consisted of 75 patients, 18 of whom were excluded (mostly because the surgical procedure they underwent was not included in RACHS-1); thus, a total of 57 patients were included in the present analysis. The characteristics of the sample are shown in table 1.

**Table 1:** Sample characteristics

Characteristic	
Age (months)	7.0 (2.0 - 17.0)
Male sex	31 (54.4)
Birth weight (kg)	3.0 (2.5 - 3.4)
Term gestational age	30 (62.5)
Z-score (BMI-for-age)	
Eutrophic	11 (31.4)
Underweight	16 (45.7)
Extremely underweight	8 (22.9)
Congenital heart disease	
AVSD + abnormal communication	1(1.8)
Complex cardiomyopathy	6 (10.5)
Aortic coarctation	6 (10.5)
Interatrial communication + interventricular communication	8 (14.0)
Fallot's tetralogy	11 (19.3)
AVSD	7 (12.3)
Interventricular communication	7 (12.3)
Interatrial communication	3 (5.3)
Transposition of the great arteries	2 (3.5)
Other	6 (10.5)
ECC duration (minutes)	105.0 (82.0 - 134.0)
Length of ICU stay (days)	8.0 (5.0 - 25.0)
IMV duration (days)	3.0 (0.3 - 8.0)
RACHS-1 category	
> 3	27 (47.4)

BMI - body mass index; AVSD - atrioventricular septal defect; ECC - extracorporeal circulation; ICU - intensive care unit; IMV - invasive mechanical ventilation. The results are expressed as the median (25 - 75% interquartile range) or n (%).

Comorbidities were observed in 59.6% of the sample, and Down syndrome with or without other comorbidities and prematurity were the most frequent (12.3%). Other syndromes were present in 5.3% of patients, and malformations were present in 3.5%. Postoperative complications were present in 87.7% of patients; the respiratory and renal systems were the most frequently involved, affecting 40.4% and 35.1% of the sample, respectively. Sepsis was diagnosed in 33.3% of the patients, pneumonia in 19.3%, and atelectasis in 14%.

**Figure 1:**
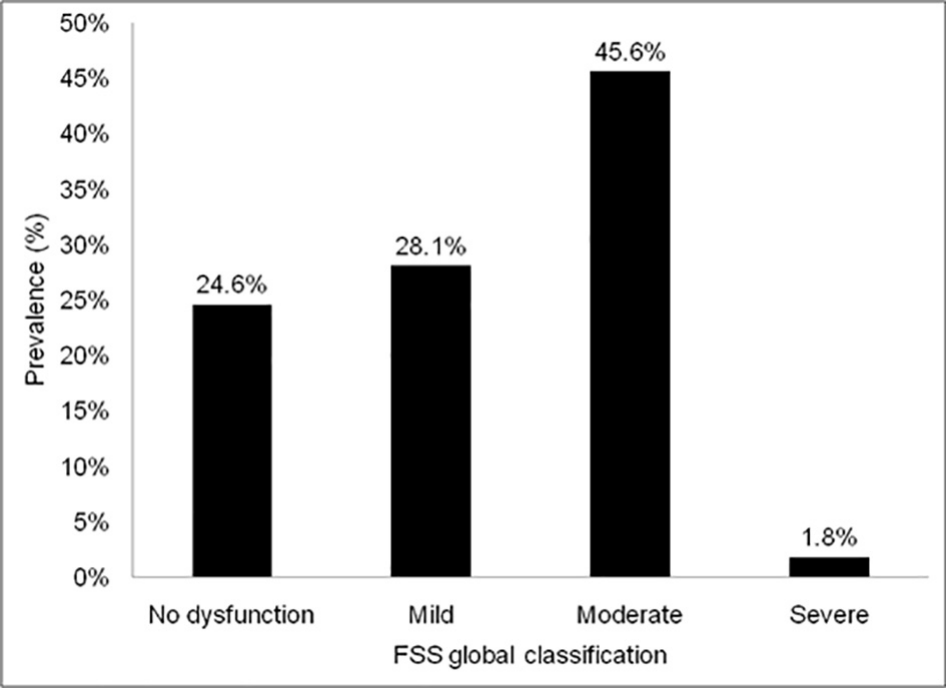
Degree of dysfunction after discharge from the pediatric intensive care unit. FSS - Functional Status Scale.

The functional outcome after discharge from the pediatric ICU, analyzed in terms of the degree of dysfunction, is shown in figure 1. We observed a high prevalence of functional deficit, with the mild and moderate degrees being the most prevalent. No patient presented very severe dysfunction.

A positive correlation was observed between surgical risk, duration of ECC, length of stay in the pediatric ICU and duration of IMV in the postoperative period and the total functional score (Table 2), demonstrating that an increase in each of these variables was associated with a worse functional prognosis. Age had an inverse correlation, indicating that younger children had worse functional status.

**Table 2:** Correlations between functional status and clinical variables

Variable	FSS total score	
	Correlation coefficient*	p value
Age	-0.483	< 0.001
RACHS-1	0.437	0.001
ECC duration	0.359	0.018
Postoperative length of ICU stay	0.648	< 0.001
Postoperative IMV duration	0.683	< 0.001

FSS - Functional Status Scale; RACHS-1 - Risk Adjustment for Congenital Heart Surgery-1; ECC - extracorporeal circulation; ICU - intensive care unit; IMV - invasive mechanical ventilation.

* Spearman.

Analyzing functional status in the dichotomized form, 30 patients had adequate function/mild dysfunction, and 27 had moderate/severe dysfunction. We found no difference between the groups in regard to gestational age: 8 (38.1%) patients were born preterm and 13 (61.9%) were born at term in the group with adequate function/mild dysfunction compared to 10 (37.0%) premature infants and 17 (63.0%) term infants among the patients with moderate/severe dysfunction (p = 0.940). In the group with adequate function/mild dysfunction, 9 patients (33.3%) were classified as RACHS-1 category > 3 compared to 18 patients (66.7%) in the group with moderate/severe dysfunction, and the difference between the groups was significant (p = 0.006).

**Figure 2:**
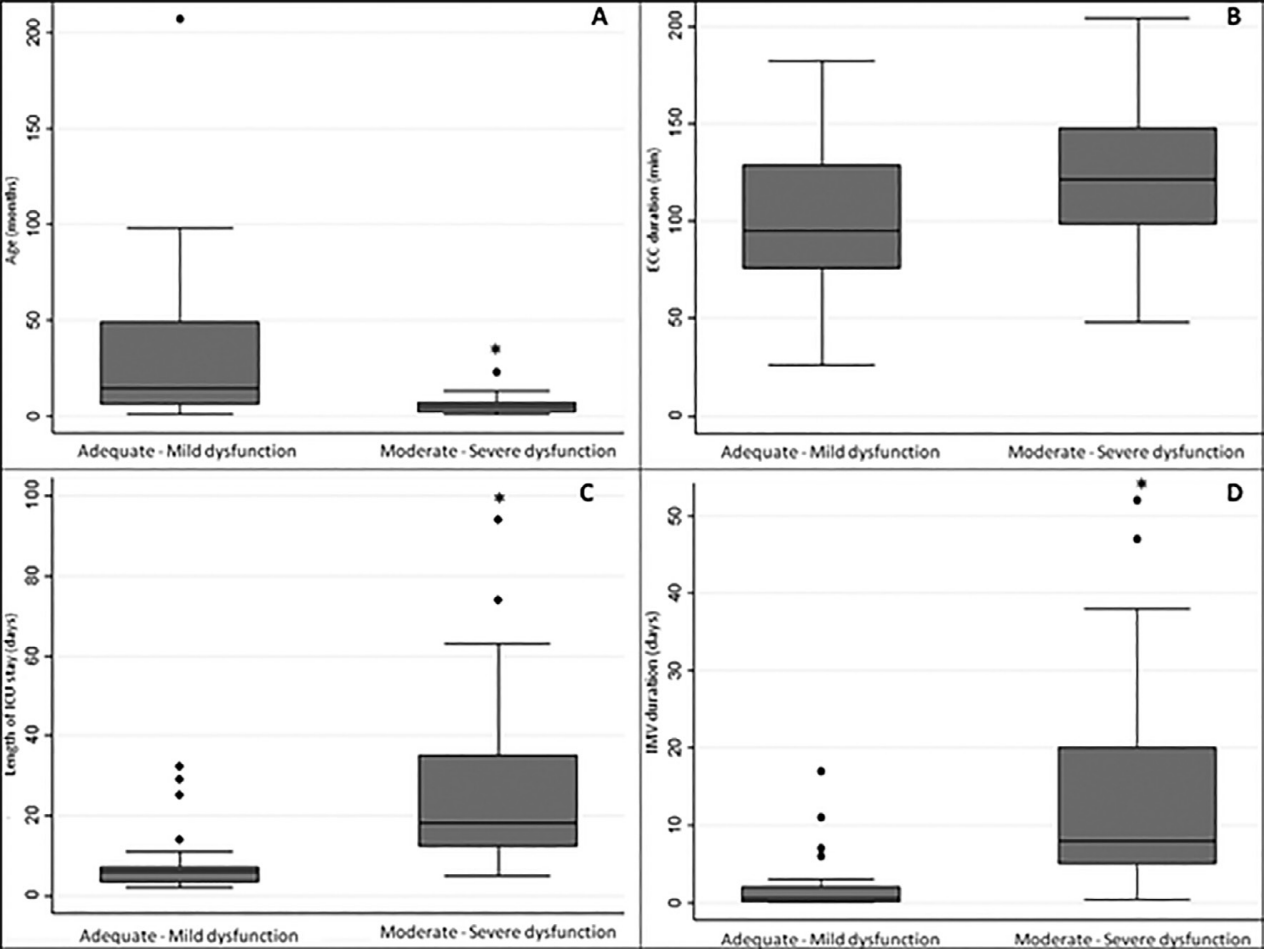
Clinical variables according to dysfunction groups. (A) Age in months; *p = 0.001; (B) duration of extracorporeal circulation in minutes; (C) length of intensive care unit stay in days; *p < 0.001; (D) duration of invasive mechanical ventilation in days; * p < 0.001. ECC - extracorporeal circulation; ICU - intensive care unit; IMV - invasive mechanical ventilation.

Figure 2 shows the clinical variables according to functional categories. The median age was 14.5 (6 - 49) months in the adequate function/mild dysfunction group and 5 (2 - 7) months in the moderate/severe dysfunction group (p = 0.001). Duration of ECC was similar between the groups (p = 0.114): 95 (75 - 129) minutes for the patients with a better functional outcome and 121 (98 - 148) minutes for the patients with a worse functional outcome. The length of stay in the pediatric ICU was higher in the moderate/severe dysfunction group than in the group with adequate function/mild dysfunction: 18 (12 - 35) days *versus* 2 (3 - 7) days, respectively; p < 0.001. Regarding the duration of IMV in the postoperative period, there was also a significant difference between groups: the group with a better functional outcome was on IMV for 0.52 (0.08 - 2.0) days, and the group with worse functional outcome was on IMV for 8 [5 - 20] days (p < 0.001).

## DISCUSSION

The development of morbidities after a stay in the pediatric ICU has been reported in previous studies,^(3,14,15,20,21)^ but for a wide range of diseases/situations. Considering this scenario, the present study is the first to investigate the functional status of pediatric patients with congenital heart disease who have undergone surgery and were admitted to the pediatric ICU using the FSS-Brazil. Dysfunction was observed in the majority of the sample, and a moderate degree of dysfunction was the most prevalent. In addition, worse functional status was observed among younger patients with a higher surgical risk according to the RACHS-1, a longer IMV duration and a longer stay in the pediatric ICU after surgery.

The high prevalence of moderate dysfunction found in the present study corroborates the results of previous studies.^(14,15)^ Pereira et al. applied the FSS after discharge from a Brazilian pediatric ICU and found that most of the sample had moderate dysfunction.^(14)^ Similarly, the study by Pollack et al. showed that a moderate degree of dysfunction was the most prevalent in patients soon after discharge from an American pediatric ICU. However, at the time of hospital discharge, there was an improvement in the functional deficit.^(15)^ Although the studies by Pereira et al. and Pollack et al. have results similar to ours, it is important to note that both analyzed patients who were admitted to the pediatric ICU for various reasons, and in the present study, we included only patients in the postoperative period of cardiac surgery.

The literature shows that after a stay in the pediatric ICU, children present important functional impairment, regardless of their underlying diagnosis. Possible risk factors for functional deficit include the pediatric ICU environment, which is poorly conducive environment to child development; the intensive monitoring; and the constant noises and light.^(27)^ In addition, the presence of drains, catheters and tubes, excessive use of sedatives, and the children’s fragile condition limit patients’ exploration of the environment and of their physical potential.^(28)^

In the present study, a higher RACHS-1 category correlated negatively with the child’s functional performance, although this correlation was weak. Previous studies have shown that cardiac surgery is a risk factor for the development of functional deficits.^(5.15)^ Berger et al.^(5)^ found that the higher the RACHS-1 category was, the higher the functional deficit rate, which may vary from 1.8% to 13.9% for those in the highest category. In a study of pediatric patients with congenital heart disease undergoing surgery, Polito et al.^(25)^concluded that a higher RACHS-1 category represented a higher risk of needing IMV for a period equal to or greater than 7 days. This finding indicates that factors related to cardiac surgery, such as its degree of complexity, affect the clinical care and interventions that children require during the postoperative period and may result in increased morbidity and significant functional decline.

Clinical variables such as the length of pediatric ICU stay and the duration of IMV after surgery were moderately correlated with worse functional performance, showing the increased impact of these factors on the functional status of children. Similar to our findings, Bone et al.^(29)^ found three risk factors for the development of functional deficits: emergency admission, longer time on IMV and longer length of pediatric ICU stay. Using the Pediatric Overall Performance Category (POPC) scale, they demonstrated a 23% incidence of dysfunction in patients with these risk factors, compared to an incidence of only 8.3% in the group that did not have these conditions.

Age also showed a weak correlation with functional outcome, with younger individuals presenting a higher risk of dysfunction.^(3,15)^ In younger children, the body systems, such as the respiratory system, are immature and fragile. In addition, neonates naturally present lower antioxidant and body self-regulation capacity, predisposing certain systems to injury, especially when associated with major heart surgery. The neurological system may be negatively affected given the changes that occur in brain perfusion and oxygenation.^(30)^ These factors may contribute to a more complicated postoperative period considering the lower efficiency and availability of children’s body mechanisms, which hinder faster recovery after physical stress and lead to a longer duration of IMV and, consequently, a longer stay in the pediatric ICU.

We found a correlation between the duration of ECC and the FSS-Brazil total score, but the correlation coefficient was weak. Children who underwent ECC for longer exhibited worse functional status due to the influence of ECC on the duration of IMV, the length of pediatric ICU stay and complications.^(31,32)^ In the present study, none of the dysfunction groups showed differences in ECC duration. This finding may be related to the fact that when we dichotomized the dysfunction variable, the ECC duration was very similar between the groups, with little variability.

The present study has some limitations, such as the lack of comparison of the functional status of the sample before and after surgery; thus, it is not possible to establish the incidence of functional deficits. However, because it was a cross-sectional study, this limitation is associated with the nature of the study only.

## CONCLUSION

Moderate dysfunction was the most prevalent degree of dysfunction in the pediatric population discharged from the pediatric intensive care unit after undergoing surgery for congenital heart disease. Possible risk factors for functional deficits were surgical risk, younger age, and longer invasive mechanical ventilation duration and length of pediatric intensive care unit stay. Considering the accelerated development and growth of children, future studies monitoring the progression of children’s functional status after pediatric intensive care unit discharge are needed to guide the implementation of early intervention plans.
